# Predictive value of erythrocyte sedimentation rate and C-reactive protein in Behcet's disease activity and manifestations: a cross-sectional study

**DOI:** 10.1186/s41927-021-00241-z

**Published:** 2022-02-11

**Authors:** Amirhossein Parsaei, Soroush Moradi, Maryam Masoumi, Fereydoun Davatchi, Anahita Najafi, Ashkan Mohammadi Kooshki, Abdolkarim Hajighadery, Massoomeh Akhlaghi, Tahereh Faezi, Hoda Kavosi

**Affiliations:** 1 Non-Communicable Diseases Research Center, Tehran University of Medical Sciences, Tehran, Iran; 2 Clinical Research of Development Center, Shahid Beheshti Hospital, Qom University of Medical Sciences, Beheshti Blvd, PO: 3719964797, Qom, Qom Iran; 3 Behcet’s Disease Unit, Rheumatology Research Center, Shariati Hospital, Tehran University of Medical Sciences, Tehran, Iran; 4 Faculty of Medicine, Tehran University of Medical Science, Tehran, Iran; 5 School of Medicine, Iran University of Medical Science, Tehran, Iran

**Keywords:** Behcet’s disease, Vascular, ESR, CRP, Autoimmune disease

## Abstract

**Background:**

Behcet’s disease (BD) as a chronic inflammatory condition that affects the eyes, skin, central nervous system, gastrointestinal tract and vessels. According to the literature, the exact value of C-reactive protein (CRP) and erythrocyte sedimentation rate (ESR) in predicting active manifestations of BD remains controversial. In this study, we aim to assess and compare values of ESR and CRP between BD patients with active/inactive BD and active/inactive manifestations of the disease. Moreover, we try to determine the predictive value of ESR and CRP for disease activity.

**Methods:**

Participants (n = 514) were drug-naïve BD patients; Based on last two visits, ESR and CRP values, disease activity, and active manifestations were recorded. The Man-Whitney U test measured the associations, and the binomial logistic regression evaluated the predictive value of ESR and CRP for active disease and each active manifestation. The sensitivity and specificity and the area under the curve (AUC) for each model were determined using receiver operating characteristic curves (ROC). Multiple regressions were run to predict BD activity score from ESR and CRP.

**Result:**

Patients with active oral, genital, joint and dermal manifestations had higher ESR and CRP values (Mann–Whitney U test, *p* < 0.05 for all). Binomial logistic regressions showed that ESR had valuable predictive value for active BD (OR = 1.09 [1.04–1.13], AUC = 0.79 [0.74–0.83], *p* < 0.001) and active vascular manifestations (1.03 [1.01–1.05], AUC = 0.85 [0.79–0.92], *p* < 0.001). CRP had good predictive value for active vascular manifestations (OR 1.98 [1.45–2.72], AUC = 0.86 [0.8–0.91], *p* < 0.001). The optimal value of ESR ≥ 10.5 and ESR ≥ 42.5 could predict active BD and active vascular manifestations with sensitivity, specificity = 71%, 75% and = 81%, 83% respectively.

**Conclusions:**

ESR and CRP are both associated with active BD and most manifestations of the diseases. They can be used for the prediction of active BD and active vascular manifestations in BD patients. Further studies can help to confirm the findings of the current research.

## Background

Behcet's disease (BD) is a chronic inflammatory condition that compromises body vessels regardless of size [1, 2]. Although ocular manifestations are the most common, BD usually involves multiple systems in the human body [3]. This unique size-independent vasculitis often has three main symptoms, including uveitis, recurrent oral ulcers, and genital sores [4]. The central nervous system (CNS) and also gastrointestinal (GI) tract could also be affected [5–7]. BD patients are at risk of thrombotic vascular involvement due to potential endothelial injuries and altered platelet functions [8, 9]. Till now, several diagnostic criteria have been developed for BD diagnosis, including International Study Group (ISG) [10] and International Criteria for Behcet's Disease (ICBD) [11]. These criteria are based on patients' clinical presentations, and no paraclinical variable is involved [12].

Different laboratory findings like complete blood count (CBC) components and cytokines have been assessed in literature to identify BD patients and predict its activity and outcome [13–15]. Both Erythrocyte Sedimentation Rate (ESR) and blood levels of C-Reactive Protein (CRP) are elevated in various infectious and inflammatory conditions, like systemic lupus erythematosus and rheumatoid arthritis [16, 17]. Although BD’s true pathophysiology is still obscure, T-cell hypersensitivity plays a key role in activating immune respone [18]. Inflammatory response provokes the release of CRP by hepatic cells [19]. As an acute phase reactanting agent, CRP binds to specific antigens in the site of inflammation and accelerates immune system activity [20]. Tissue damage results in fibrinogen release in the blood which leads to an increase in ESR [17].

Previous studies reported elevated serum ESR and CRP levels in BD patients compared to the normal population, but the exact role of these parameters in the diagnosis and assessment of BD remains unclear [21, 22]. Although researchers' opinion on the utility of ESR and CRP values in distinguishing active BD from inactive type is united, the difference of these indices among different manifestations of BD remains controversial. While some studies found significant increase of ESR and CRP levels in mucocutaneous and vascular manifestations [21], others observed this difference in other presentations like active ocular and gastrointestinal involvement [23].

Despite several studies on this matter, the diagnostic value of ESR and CRP for predicting BD activity and its different manifestations is yet to be determined. In this study, we investigated ESR and CRP values in BD patients and compared these values in different manifestations of the disease to evaluate these simple, inexpensive tests in the diagnosis of BD and predicting its possible outcomes.

## Methods

### Study design and participants

This is a cross-sectional, observational, single-center study conducted between January 2017 and January 2021 at a tertiary medical center (Shariati Hospital), Tehran, Iran. The study protocols were designed according to the Strengthening the Reporting of Observational Studies in Epidemiology (STROBE) statement and it is approved by the Tehran University of medical sciences ethics committee.

We recruited a consecutive sample of patients who had diagnosed with BD from the Shariati hospital rheumatologic clinic, regardless of their current disease activity. Only drug-naive patients enrolled in the study and patients with other inflammatory or autoimmune diseases, including recent infections, endocrine disorders, and malignancies, were excluded. In cases with clinical suspicion of infectious diseases that may affect the levels of ESR and CRP, a second evaluation by an infectious diseases specialist was conducted. The patients who had one of the mentioned conditions were excluded according to the consultation and evaluation by the second specialist. The study population only included patients with definite BD, who had no active infection, inflammatory conditions, endocrine disorders or malignancies. Informed consent was taken from all participants. An expert rheumatologist diagnosed the BD based on the International Criteria for Behçet's Disease (ICBD) and assessed activity of disease with Iranian Behçet's Disease Dynamic Activity Measurement (IBDDAM) based on active manifestations in last two visits. Patients’ demographic and clinical data including age, gender, and disease duration, were recorded along with the IBDDAM score questionnaire.

### Defining the active or inactive BD, and IBDDAM calculation

We divided participants into two categories as active/inactive BD patients based on active manifestations of the last two visits. In the current study, participants with one or more active manifestation are considered active BD patients.

The IBDDAM score is a quantitative measure for BD activity and is calculated based on the patient's clinical manifestations severity and duration. Ten clinical manifestations are assessed as follows: (1) *Oral aphthosis*: every five aphthous lesions gain one point. (2) *Genital aphthosis*: every lesion, one point. (3) *Skin lesions*: pseudofolliculitis, every ten lesions, one point; Erythema nodosum, every five lesions, one point. (4) *Ocular lesions*: anterior uveitis, 1–4 points are given for flare, hypopyon, cell, and keratic precipitate; Posterior uveitis, 1–4 points are given for cell, snowball, and snow banking, and the total is multiplied by two (gravity indices); Retinal vasculitis: 1–4 points is given for edema of the disk, macula edema, and retinal edema, periphlebitis, periarteritis, and papillitis. The total is multiplied by three (gravity index). Visual acuity is calculated by the Snellen chart. The observed number is subtracted from 10, and the remaining is multiplied by two (gravity index). As an example, visual acuity of 8/10 would give (10 − 8) * 2 = 4. The score is calculated separately for each eye. (5) *Joints*: arthritis: Arthralgia, one point. Monoarthritis, two points. Polyarthritis, three points. (6) *Central nervous system (CNS) involvement*: Mild headache, one point. Mild CNS involvement, three points. Moderate to severe CNS manifestation, six points. (7) *Vascular involvement*: Superficial phlebitis, one point. Deep vein thrombosis (each vein), two points. Large vessel involvement (each vessel), six points. (8) *Gastrointestinal tract*: Mild manifestations, three points. Moderate to severe manifestations, and six points. (abdominal pain,chronic diarrhea and rectal bleeding). (9) *Epididymitis*: two points. (10) *Positive pathergy test*: one point. Duration of lesions: if a lesion does not resolce in one month, the same points are cosidered for each more month.

### Laboratory data

Blood samples were taken during the last two visits and were analyzed to determine the erythrocyte sedimentation rate (ESR), and C- reactive protein (CRP). Laboratory tests were performed for each sample, and the average of two findings was reported.

### Bias

The risk of bias for the clinical judgment was limited by clinician's judgment according to ICBD criteria and the IBDDAM scoring system. Laboratory tests were also analyzed in an exclusive laboratory in Shariati hospital.

### Statistical analysis

Continuous variables were demonstrated as mean ± standard deviation (SD), and categorical variables were described in count and percentage. intial analyses did not show outliers, as assessed by a boxplot. The variables were also tested for normal disrtibution with the Shapiro–Wilk's test; since ESR was a continuous variable without a normal distribution (Shapiro–Wilk's test's *p*-value < 0.05) and CRP was an ordinal variable (ranging 0–4), Mann- Whitney U test was used to analyze data; The number of patients with epididymitis, CNS and GI manifestations of BD were insufficient to fulfill the Man-Whitney U test assumption of equal distribution (*p*-value of Levene's test for equality of variances based on median and with an adjusted degree of freedom < 0.05).

Binomial logistic regression was utilized to evaluate the predictive powers of ESR and CRP for active BD and its each active manifestation separately. The Hosmer and Lemeshow test was used to assess the fit of the risk prediction models; and for the logit of the dependent variable, the linearity of continuous variables was assessed using the Box–Tidwell statistics. Fore each model, the sensitivity, specificity and also area under the curve (AUC) were determined with receiver operating characteristic (ROC) curves. To evaluate the optimal cut-off values for our dichotomous diagnostic test Youden's J statistics were utilized.

For further prediction, we used multiple regressions to predict the activity score (IBDDAM) from values of ESR and CRP. Linearity was observed in partial regression plots and also a plot of residuals versus the predicted values. The residuals independence was confirmed using Durbin–Watson statistic. The homoscedasticity was verified by inspection of a plot of residuals against unstandardized predicted values. The tolerance values were higher than 0.2 and no evidence of multicollinearity was found. The statistical analysis was done using SPSS version 26 (SPSS Inc. Chicago, IL). A *p*-value < 0.05 was statistically significant in all tests.

## Results

### Patients

Five hundred fourteen patients with a history of BD between 11 and 70 years old (mean age 36.5 ± 12 years) were enrolled. Of these, 220 patients (42.8%) were female and 294 (57.2%) were male. Average values for Erythrocyte Sedimentation Rate (ESR) were 26.3 ± 23.4; 323 patients (62.8%) had negative C-Reactive Protein (CRP) results and the other 191 patients had positive CRP results with average values of 2.2 ± 1. The prevalence of BD manifestations is summarized in Table 1; Four hundred fifty patients (85%) had active BD manifestations in their latest examination.

**Table 1:** Erythrocyte sedimentation rate (ESR) and C-reactive protein (CRP) values based on active manifestations of Behçet’s disease

BD active manifestations	Count (%)	ESR (mean ± SD) mm/hr	*p*-value	CRP (mean ± SD) mg/L	*p*-value
*Active BD*					
Yes	450 (87.5%)	28.9 ± 23.8	< 0.001*	0	< 0.001*
No	64 (12.5%)	7.8 ± 4.5		0.9 ± 1.2	
*Active ocular signs*					
Yes	234 (45.5%)	20.7 ± 18.4	< 0.001*	0.6 ± 1	< 0.001*
No	280 (54.5)	30.9 ± 26		1 ± 1.3	
*Active oral signs*					
Yes	254 (49.4%)	35.2 ± 23.9	< 0.001*	1.2 ± 1.3	< 0.001*
No	260 (50.6%)	17.2 ± 19		0.4 ± 0.9	
*Active genital signs*					
Yes	99 (19.3%)	43 ± 21.7	< 0.001*	1.8 ± 1.2	< 0.001*
No	415 (80.7%)	22.3 ± 22		0.6 ± 1.1	
*Active vascular signs*					
Yes	36 (7%)	60.4 ± 20.2	< 0.001*	2.6 ± 1.1	< 0.001*
No	478 (93%)	23.7 ± 21.1		0.7 ± 1.1	
*Active joint signs*					
Yes	68 (13.2%)	50.2 ± 20.2	< 0.001*	1.8 ± 1.2	< 0.001*
No	446 (86.8%)	22.6 ± 21.6		0.6 ± 1.1	
*Active dermal signs*					
Yes	180 (35%)	36.7 ± 20.2	< 0.001*	1.4 ± 1.3	< 0.001*
No	336 (65%)	20.7 ± 23		0.5 ± 1	
*Positive pathergy test*					
Yes	106 (20.6%)	23.3 ± 25.5	0.017*	0.6 ± 1.2	0.011*
No	408 (79.4)	27 ± 22.8		0.8 ± 1.2	
Epididymitis**					
Yes	6 (1.2%)	50.1 ± 16.9	–	2.3 ± 1	–
No	508 (98.8%)	26 ± 23.3		0.8 ± 1.2	
Active CNS signs**					
Yes	7 (1.4%)	58.7 ± 38.5	–	1.8 ± 1.8	–
No	507 (98.6%)	25.8 ± 22.9		0.8 ± 1.2	
Active GI signs**					
Yes	2 (0.4%)	99 ± 33.9	–	3.5 ± 0.7	–
No	512 (99.6%)	26 ± 22.9		0.8 ± 1.2	
Total	514	26.3 ± 23.4		0.8 ± 1.2	

BD, Behçet’s disease; CRP, C-reactive protein; ESR, erythrocyte sedimentation rate; SD, standard deviation

*Statistically significant (Man-Whitney U test, *p*-value < 0.05)

**Insufficient number to fulfill the Mann–Whitney U test’s assumption of equal distribution

### ESR and CRP in different BD manifestations

The association of ESR and CRP with different manifestations of BD is presented in Table 1. We investigated whether differences in ESR or CRP corresponded with the various active signs of BD. Patients with active BD showed significantly higher ESR (Mann–Whitney U test, *p* < 0.001) in comparison to patients with inactive BD. Similarly, patients with oral, genital, vascular, joint and dermal manifestations had higher ESR (Mann–Whitney U test, *p* < 0.05) and CRP (Mann–Whitney U test, *p* < 0.05). Number of patients with active CNS and GI signs and epididymitis was insufficient to evaluate their association with ESR and CRP.

### Value of ESR and CRP for predicting different BD manifestations

Binomial logistic regression analyses evaluated the predictive models of ESR and CRP for active BD and its each active manifestation separately. The results are presented in Table 2. ESR or CRP considered as a valuable predictor when all of the following criteria were met: *p*-value < 0.05, Hosmer–Lemeshow's *p*-value > 0.05 and the area under the ROC curve (AUC) more than 0.7. ESR was a valuable predictor for active BD and its vascular manifestations; and also, CRP was a good predictor for active vascular manifestations. The outputs of logistic regression models indicated that every ten-unit increase of ESR increases the likelihood of active BD and active vascular manifestations, 90% and 30%, respectively. Since the number of patients with active GI or CNS manifestations or epididymitis were too low, validity of the predictive values of ESR for active GI and CNS manifestations—which fulfilled the aforementioned criteria—were not accepted. The other manifestations of BD were not well predicted by ESR or CRP. The optimal cut-off value of ESR for BD activity and vascular manifestations, using Youden's J, were 10.5 and 42.5, respectively. The sensitivity and specificity for the cut-off point of ESR for BD activity were 71% and 75%, respectively, and for active vascular manifestations were 81% and 83%, respectively.

**Table 2:** Summary of binary logistic regression models for predicting BD manifestations based on ESR and CRP

Condition	Variable	Wald	OR	95% CI	*p*-value	HS value	R square	AUC	95% CI
Active BD	ESR*	17.7	1.09	1.04–1.13	< 0.001	0.58	0.32	0.79	0.74–0.83
	CRP	0	–	–	0.99			0.71	0.66–0.76
Active vascular signs	ESR*	18.2	1.03	1.01–1.05	< 0.001	0.78	0.37	0.85	0.79–0.92
	CRP*	18.4	1.98	1.45–2.72	< 0.001			0.86	0.8–0.91
Active ocular signs	ESR	12.2	0.98	0.97–0.99	< 0.001	0.22	0.06	0.39	0.34–0.44
	CRP	0.8	0.91	0.76–1.1	0.35			0.41	0.36–0.46
Active oral signs	ESR	30.5	1.03	1.02–1.04	< 0.001	< 0.001	0.23	0.74	0.7–0.79
	CRP	11.7	1.41	1.15–1.71	0.001			0.69	0.64–0.73
Active genital signs	ESR	11.8	1.02	1.00–1.03	0.001	< 0.001	0.23	0.78	0.73–0.82
	CRP	25.0	1.67	1.36–2.04	< 0.001			0.78	0.73–0.83
Active joint signs	ESR	30.5	1.03	1.02–1.05	< 0.001	0.001	0.25	0.84	0.8–0.87
	CRP	4.9	1.29	1.03–1.63	0.02			0.74	0.67–0.8
Active dermal signs	ESR	14.3	1.02	1.00–1.03	< 0.001	< 0.001	0.17	0.74	0.7–0.78
	CRP	15.8	1.44	1.20–1.73	< 0.001			0.70	0.65–0.75
Positive pathergy test	ESR	0.2	0.99	0.98–1.00	0.61	0.04	0.01	0.42	0.36–0.48
	CRP	1.4	0.86	0.69–1.09	0.22			0.43	0.37–0.49
Epididymitis**	ESR	0.7	1.01	0.98–1.04	0.39	0.14	0.12	0.81	0.72–0.9
	CRP	2.8	1.77	0.91–3.42	0.08			0.83	0.74–0.91
Active GI signs**	ESR*	3.9	1.07	1–1.15	0.04	1	0.5	0.97	0.94–1
	CRP	0.8	2.41	0.36–16.04	0.36			0.94	0.88–0.99
Active CNS signs**	ESR*	6.0	1.03	1.00–1.06	0.01	0.77	0.14	0.77	0.59–0.95
	CRP	0.05	1.07	0.56–2.00	0.82			0.65	0.41–0.9

OR, odds ratio; CI, confidence interval; HS, Hosmer–lemeshow test; R square, Nagelkerke R square; AUC, area under the curve

*Statistically significant *p*-value with statistically non-significant Hosmer–Lemeshow test (model fitness) and area under the curve > 0.7

**Low number of patients with GI and CNS manifestations and epididymitis (0.4%, 1.4% and 1.2%, respectively) decreases the prediction model’s validity

### Predictive value of ESR and CRP for IBDDAM score

**Fig. 1:**
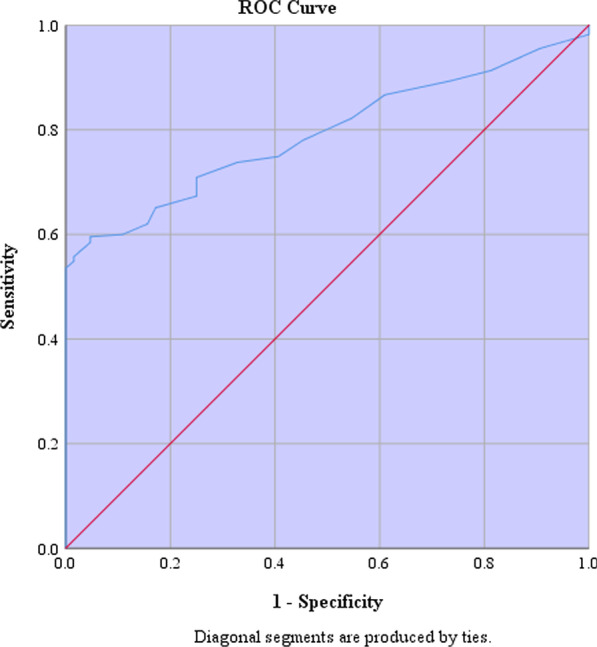
Receiver operating curve of erythrocyte sedimentation rate (ESR) in patients who had active BD

**Fig. 2:**
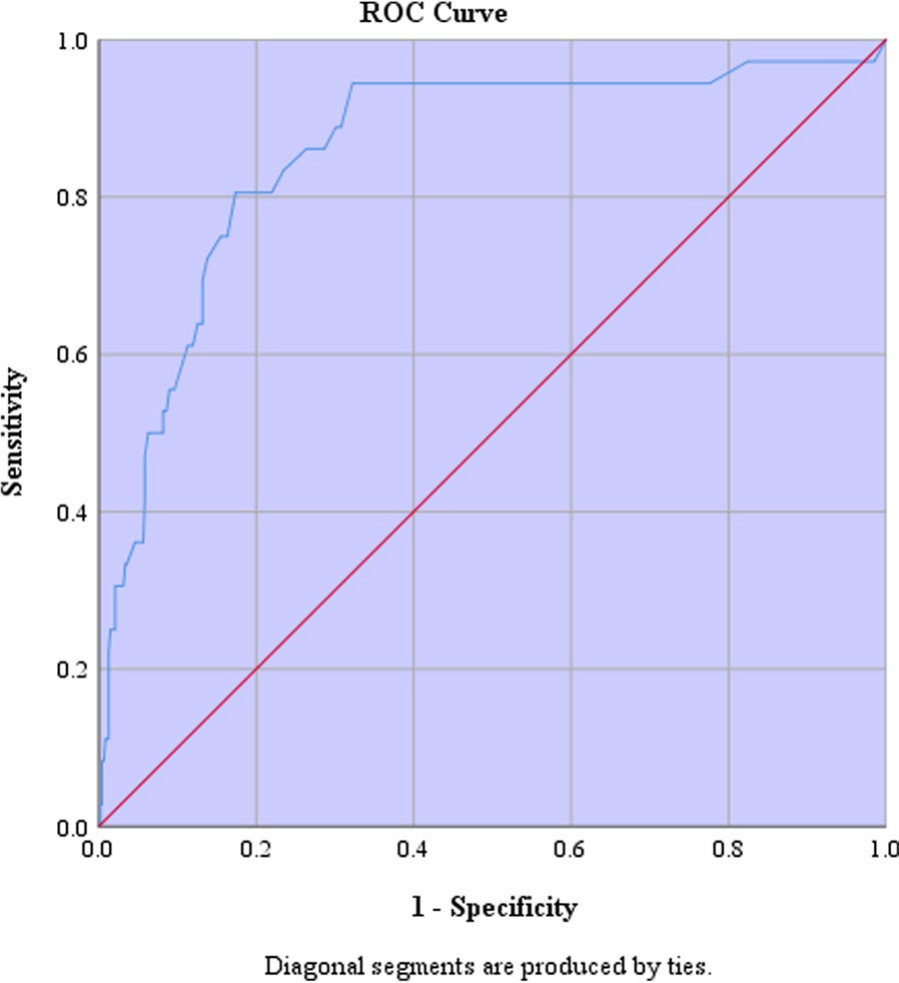
Receiver operating curve of erythrocyte sedimentation rate (ESR) in patients diagnosed with active vascular manifestations of BD

Multiple regressions analysis was used to predict IBDDAM score from the patients' age, gender, ESR and CRP measures. according to calculated the Durbin–Watson statistic of 1.81, the residuals were independent. None of the four independent variables of the multiple linear regression model was not statistically significant in predicting the patient's IBDDAM score. The regression coefficients are reported in Table 3, Figs. 1 and 2.

**Table 3:** Multiple linear regression analysis for IBDDAM score

Variable	B (CI)	*p*-value	Adjusted R square	Durbin–Watson
ESR	− 0.06 (− 0.16 to 0.02)	0.14		
CRP	− 0.82 (− 2.61 to 0.97)	0.36		
Age	− 0.12 (− 0.29 to 0.35)	0.12		
Gender	1.32 (− 2.32 to 4.98)	0.47		
Model Summary		0.07	0.009	1.81

B, Unstandardized beta coefficient; CI, Confidence Interval

## Discussion

This study assessed the association of ESR and CRP, two simple and inexpensive blood tests, with BD activity and a range of its manifestations in drug-naive patients. ESR was significantly higher in patients with active BD rather than inactive disease. ESR and CRP values were higher in patients with active oral, genital, and vascular manifestations compared to subjects with no expressions of these symptoms. In logistic regression models, predictive values of ESR showed promising results in measuring BD activity, for each ten units increase in ESR the likelihood of active BD is increased by 90% (OR: 1.09, AUC: 0.79). The sensitivity and specificity of ESR levels were 71% and 75% for prediction of active BD, and 81% and 83% for its active vascular manifestations. Both ESR and CRP indices were also reliable predictive values for active vascular manifestations of BD (OR:1.03 AUC:0.85 for ESR, and OR:1.98 AUC:0.86 for CRP). However, these paraclinical parameters were unable to predict disease activity based on IBDDAM scores.

ESR and CRP levels are elevated in various conditions, including inflammations, infections, and malignancies [24]. Despite their good sensitivity in determination of an inflammatory state, ESR and CRP's lack of specificity in differentiating the underlying cause limited their clinical application in diagnosis or assessment of a certain condition [19]. ESR remained as a tool for diagnosis or response measurement to therapy for only few diseases: polymyalgia rheumatica, temporal arteritis, and rheumatoid arthritis [25]. T-cell hypersensitivity in BD could result in activation of immunity cascade and subsequently overstimulation of innate immune system [18]. Hyperexpression of acute phase reactants like CRP which binds to desired antigens in inflammation site, as well as increased fibrinogen release from damaged tissue which delays our RBCs' sedimentation, could justify the elevation of ESR and CRP levels in BD patients [26]. Also following previous studies on CRP increase in cardiovascular event, our significant elevated ESR and CRP result in active vascular manifestations of the disease could be justified [27].

Numerous studies assessed ESR and CRP values in BD patients. Isik et al. study on 21 BD patients compared to 25 healthy controls showed significantly increased ESR and CRP values in BD patients [28]. Alli et al. also found this statistically significant but weak correlations between disease activity scores and ESR and CRP indices in a study on 213 BD patients [29]. Although none of these studies compared their results in different manifestations of BD. On the other hand, other studies that investigated these values in specific manifestations of the disease showed controversial results. In a study on 60 patients with BD, Lehner et al. found a statistically significant difference in ESR values for all disease manifestations except for neurologic symptoms [23]. However, these significant differences were only observed in patients with erythema nodusom, vascular and arthritic involvement in a study by Müftüoǧlu et al. on 150 BD patients [21].

These controversial results and lack of evidence on ESR and CRP values in BD manifestations required further investigations on this matter. Our study results not only showed significant difference in patients with active disease compared to inactive disease, but also were able to differentiate some of BD manifestations, especially vascular, based on ESR and CRP indices. Also, these simple paraclinical tools represented reliable predictive values for BD activity and its vascular symptoms.

In a study in 1986, Müftüoǧlu et al. showed significantly higher ESR and CRP results in patients with active BD rather than inactive or control group [21]. This result was also supported by Tanacan et al. study in 2020 which ESR and CRP levels in 103 active BD patients showed a statistically significant difference compared to 63 patients in the inactive disease group [30]. Our study results also confirm this hypothesis since ESR and CRP values in 450 individuals with active BD showed considerable difference compared to 64 inactive BD patients. Following our previous study results confirming serum CRP levels to be significantly higher in BD patients than in the normal population [15], the current study implies that CRP values could be further utilized to differentiate active and inactive BD behavior.

To establish a thorough connection between laboratory results and different manifestations of BD concerned researchers in recent years [31–33]. Complete blood count (CBC) test components including RDW and MPV [34], NLR and PLR [15], and various cytokines like TNF-α, IL-6 and IL-8 [35, 36] have been discussed on this matter in previous studies. Lehner et al. in a study with 60 patients with Behcet's syndrome and 34 matched controls, except for the neurological group, ESR and CRP were significantly elevated in different manifestations of BD with the highest amount in arthritic type [23]. On the other hand, Müftüoǧlu et al. found this considerable difference in erythema nodosum and acute thrombophlebitis for both ESR and CRP levels, and in arthritic type only for ESR (*p* < 0.01). Their study results showed no significant difference for ESR and CRP values in central nervous system (CNS) manifestations and, interestingly, ocular and mucocutaneous involvement. In the current study, significant differences in serum ESR and CRP levels were evident in oral, genital, vascular, arthritic, and dermal manifestations, with the highest levels for active vascular signs (*p* < 0.001). As mentioned in previous studies, our results also failed to establish a significant difference in ESR and CRP values in active CNS involvement, gastrointestinal manifestations, and patients with epididymitis. The extreme rarity of these manifestations [37], in our study 7 (1.4%) for active CNS signs, 6 (1.2%) for epididymitis, and only 2 (0.6%) for active GI involvement, could vindicate this incapability, as both Müftüoǧlu et al. and Lehner et al. believed the same [21, 23].

As a supplementary histopathologic tool for the diagnosis of BD, the Pathergy test was one of the variables our study favored to investigate [38]. In comparison between 408 (79.4%) BD patients with negative and 106 (20.6%) with positive pathergy test, ESR and CRP values were significantly higher in the latter group (*p* = 0.017 and *p* = 0.011, respectively). We investigated the overall activity of BD in our participants with the IBDDAM scale, a dynamic quantitive checklist based on manifested symptoms duration and severity [4]. Our results lack the strength to establish a solid predictive value for IBDDAM score in none of four variables investigated with multiple linear regression, including age (*p* = 0.12), gender (*p* = 0.47), serum ESR (*p* = 0.14), and CRP (*p* = 0.36) levels. The predictive value of ESR and CRP for different BD manifestations was also assessed with binomial logistic regression. This analysis revealed ESR as a reliable predictor of active BD (patients with at least one active presentation, *p* < 0.001, AUC = 0.79) and active vascular manifestations (*p* < 0.001, AUC = 0.85). CRP predictive value was also significant for vascular involvement of BD (*p* < 0.001, AUC = 0.86). The sensitivity and specificity of serum ESR levels were 71% and 75% for active BD, 81%, and 83% for active vascular manifestations.

We had several limitations in the course of this study. Although ESR and CRP results showed promising correlations with BD activity and its different presentations, our cross-sectional study investigated samples from a single tertiary center retrospectively. The value of these findings could be evaluated with future prospective cohort studies on a database from multiple centers. Our analysis was unable to measure the difference of ESR and CRP values in some manifestations of BD, like active CNS or GI involvement, due to insufficient samples. We recommend future studies investigating these parameters in BD patients with active CNS symptoms, epididymitis, or active GI presentations. Since ESR and CRP values are affected by several conditions, we suggest future studies to investigate these parameters for longer periods in patients using medical treatment or facing other comorbidities which could give us a better understanding of confounding factors affecting ESR and CRP levels. Since the current study excluded patients with active infection or other conditions like inflammatory diseases, the predictive value of ESR and CRP for BD activity is limited and needed to be further evaluated in these conditions (such as complicated infections). We also like to suggest the investigation of different and symptoms' intensity and possible changes in these values.

In conclusion, serum ESR and CRP levels are significantly higher in patients with active BD and its different active manifestations. These two routinely used inexpensive tests also showed a reliable predictive value for active BD disease and active vascular involvement. However, future studies are necessary to validate this matter.

## Data Availability

The datasets that are used in the current study are available from the corresponding author upon reasonable request by researchers.

## Abbreviations

AUC: Area under the curve
BD: Behcet’s disease
CBC: Complete blood count
CNS: The central nervous system
CRP: C-reactive protein
ESR: Erythrocyte sedimentation rate
GI: Gastrointestinal
IBDDAM: Iranian Behçet’s Disease Dynamic Activity Measurement
ICBD: International Criteria for Behcet’s Disease
ISG: International Study Group
ROC: Receiver operating characteristic curves
STROBE: Strengthening the reporting of observational studies in epidemiology
